# Brain-Derived Neurotrophic Factor and Oxytocin Signaling in Association With Clinical Symptoms in Adolescent Inpatients With Anorexia Nervosa—A Longitudinal Study

**DOI:** 10.3389/fpsyt.2019.01032

**Published:** 2020-02-28

**Authors:** Marta Tyszkiewicz-Nwafor, Filip Rybakowski, Monika Dmitrzak-Weglarz, Maria Skibinska, Elżbieta Paszynska, Agata Dutkiewicz, Agnieszka Słopien

**Affiliations:** ^1^Department of Child and Adolescent Psychiatry, Poznan University of Medical Sciences, Poznan, Poland; ^2^Department Psychiatry, Poznan University of Medical Sciences, Poznan, Poland; ^3^Department of Psychiatric Genetics, Poznan University of Medical Sciences, Poznan, Poland; ^4^Department of Integrated Dentistry, Poznan University of Medical Sciences, Poznan, Poland

**Keywords:** eating disorders, anorexia nervosa, brain-derived neurotrophic factor, oxytocin, tropomyosin-related kinase B

## Abstract

**Introduction:**

Brain-derived neurotrophic factor (BDNF), as well as oxytocin (OXY), are centrally secreted neuropeptides regulating a range of physiological processes, including food intake and metabolism. Moreover, numerous reports suggest their role in affective and cognitive symptoms of various psychiatric disorders. Thus, the study aimed to measure the serum level of BDNF and its receptor—tropomyosin-related kinase B (TrkB) and OXY in the malnourished anorexia nervosa patients and following partial weight-recovery. The correlations between levels of these proteins with the primary symptoms of the anorexia nervosa (AN) were also analyzed.

**Methodology:**

Eighty-four adolescent AN patients were recruited into the study, but only forty-two AN patients completed it. The control group comprises of thirty age- and height-matched girls (CG). Serum BDNF, TrkB, and OXY levels were measured in AN group in two time-points—at the beginning of the hospitalization in malnourished patients (AN-T1) and again after partial weight normalization, on the day of discharge (AN-T2). The severity of eating disorders, as well as depressive and obsessive-compulsive symptoms, were assessed at the same two-time points.

**Results:**

Body mass index (BMI) differed significantly between the AN-T1, AN-T2, and CG. BDNF levels for the AN-T2 increased significantly in comparison to the AN-T1, but at two-time points were significantly lower than in the CG. The OXY level did not change with weight gain and in both groups AN-T1 and AN-T2 were statistically significantly higher than in the CG. Statistically significant negative correlations between BDNF and the severity of eating disorders symptoms were found. Depressive and obsessive-compulsive symptoms did not show significant correlations with levels of studied proteins for either malnourished or partially weight recovered AN patients.

**Conclusions:**

BDNF serum levels were decreased in the malnourished AN patients and tended to normalize with partial weight recovery. OXY serum levels were found to be increased in the malnourished AN patients and did not normalize with partial weight recovery, confirming previous reports about its role in the etiopathogenesis of AN. BDNF can be related to aberrant eating behaviors occurring in AN. Our results do not support the role of serum levels of BDNF, TrkB, or OXY in the modulation of depressive or obsessive-compulsive symptoms.

## Introduction

Anorexia nervosa (AN) is common (1), poorly understood metabo-psychiatric disorder with a high relapse rate (2) and excessive mortality (3). Biological, psychological, and sociocultural factors may affect the development, progression, and outcome of AN, and recently a role for the abnormalities in centrally and peripherally produced regulatory peptides in the AN pathogenesis has been suggested (4). Regulatory proteins play an essential role in the monitoring of food intake, affecting homeostatic, mainly hypothalamic control of feeding. Moreover, they may also influence non-homeostatic food intake *via* their receptors in the cortico-limbic system. Processes like emotions, motivation, physical activity and reward assessment, associated with higher order brain structures are also relevant for the regulation of food intake and AN etiology (5).

Oxytocin (OXY) is a nine-amino acid neuropeptide and neuromodulator mainly produced in the paraventricular nucleus (PVN) (6) and the supraoptic nucleus (SON) (7), which primarily regulates reproductive behaviors and mother–infant interactions (8). It is released into the bloodstream, entering peripheral circulation, as well as directly into the nervous system (9). In the brain, OXY binds to a G-protein-coupled receptor expressed in the limbic system so its role in learning, anxiety, memory, regulation of emotions, and social cognition has been suggested (10). Moreover, its receptors are also present peripherally in pancreas, gastrointestinal tract, or adipocytes (11), which may point to its importance for metabolism and food intake (12). In animals, OXY-deficiency or OXY receptor-deficiency increased food intake and body weight (13). Moreover, central or peripheral OXY administration attenuated food intake and led to sustained weight reduction (14). It was also demonstrated that OXY activates pro-opiomelanocortin neurons (POMC) and acts as the downstream mediator of the leptin effects. Peris et al. proposed that the peptide might attenuate eating behavior by modulating reward-related signaling (15). Serotonin has been found to increase OXY concentrations (16), while dopamine and glutamate interactions with OXY were found to modulate the activity of the reward circuitry of the brain (15). The serum OXY levels in obese patients were found to be increased (17). However, if obesity was associated with diabetes then the serum OXY levels were decreased (18). Moreover, in both healthy-weight and obese subjects, OXY administration can cause a reduction in caloric intake and consumption of palatable snacks (19), as well as improved glucose and lipid metabolism (20, 21). OXY increased the cognitive control and reduced food craving, which might suggest its role in the reward system (22). Thus, the above results support the interest in the role of OXY as an anorexigenic factor both in healthy humans and patients suffering from eating disorders.

Brain-derived neurotrophic factor (BDNF) is produced in the brain and plays an essential role in neuronal survival and growth, serves as a neurotransmitter and neuromodulator, and participates in neuronal plasticity (23). BDNF protein and its receptor tropomyosin-related kinase B (TrkB) have been identified in most brain areas including the olfactory bulb, cortex, hippocampus, basal forebrain, mesencephalon, hypothalamus, brainstem, and spinal cord (24). Furthermore, it was shown that BDNF is involved in the regulation of food intake and metabolism and, together with TrkB, is expressed in several regions that influence feeding behavior including the PVN, arcuate nucleus (ARC), ventromedial nucleus (VMN), dorsomedial hypothalamus (DMH), and lateral hypothalamus (LH) (25, 26). BDNF affects the motivated and reward-seeking behaviors in the mesolimbic dopamine system (27, 28) and thus regulate the food intake (29). Animal studies have shown that BDNF or TrkB depletion results in hyperphagia, obesity, and metabolic syndrome (30). Moreover, peripheral or ventricular administration of BDNF suppresses energy intake and reduces body weight (31). In humans, BDNF haploinsufficiency or inactivating mutations of the BDNF receptor result in the hyperphagia and childhood-onset obesity (32, 33). Moreover, large genome-wide association studies have strongly implicated the BDNF gene locus in the regulation of body mass index (BMI) (34). It was postulated that BDNF could have an anorexigenic effect on food intake and metabolism.

The majority of previous studies examined the role of OXY, BDNF, and TrkB in adult patients with AN. It is not clear whether the regulation of food intake in the developing adolescents is the same as in adults. Nevertheless, the assessment of the adolescent population may allow to observe the regulatory mechanisms before the neuroplastic changes caused by long duration of illness.

Therefore, the presented study aimed to measure the serum levels of OXY, BDNF, and TrkB in malnourished and partially weight-recovered adolescent AN inpatients. We investigated the correlations between level of proteins and several symptomatic dimensions of AN, namely eating disorder, depression, and obsessive-compulsive symptoms. Due to the lack of previous studies in this population, unclear results of animal models, and exploratory nature of the project no *a priori* hypotheses of correlation were proposed.

## Methodology

### Participants and Procedures

Eighty-four adolescent patients admitted in the acute phase of AN to the Child and Adolescent Psychiatric Department were enrolled in the study. Diagnosis of the restrictive type of AN was made according to the ICD-10, DSM-IV, and DSM-5 criteria after a semistructured interview conducted by a child and adolescent psychiatrist. A physical examination and basic laboratory assessments were performed. The exclusion criteria comprised physical illnesses and laboratory abnormalities not resulting from the prolonged restriction of food as well as other psychiatric comorbidities. For the first time during the study, in 1 to 3 days after admission in malnourished AN patients (AN-T1), a psychometric assessment was conducted, heights and weights were checked, and 15 ml blood samples were obtained. The same procedures were repeated for the same patients in the second time point 11.2 ± 2.3 weeks later, on the day of discharge, after partial weight normalization (AN-T2). Then the exclusion criteria were as follows: failure to obtain target body weight (minimal appropriate for particular height and age) and an increase of BMI less than 2 kg/m^2^. Only forty-two weight-recovered amenorrhoeic patients completed the study, and the remaining forty-two subjects who did not gain weight or discharged themselves against medical advice were excluded. The control group (CG) comprised of thirty normal-weight, eumenorrheic, healthy girls with no history of psychiatric disorders that were recruited from middle school students. They underwent the same procedures as physical and psychiatric examination, anthropometric and psychometric assessment, and blood analysis, only once. The BMI was calculated as a ratio of body weight (kg) to height (m^2^) and the percentage of ideal body weight (%IBW) as a ratio of actual to ideal body weight (IBW) × 100%, where IBW (kg) = [height (cm)−100]−{[height (cm)−150]/2} according to Lorentz’s formula. Considering Polish growth references, the additional inclusion criterion was a BMI of lower than 15 kg/m^2^ (35). All measurements were taken from fasting females in a standing position. The Eating Attitudes Test (EAT-26), Hamilton Depression Rating Scale (HDRS), Beck Depression Inventory (BDI), and Yale-Brown Obsessive-Compulsive Scale (YBOCS), all commonly used in clinical practice as well as scientific research in the Polish adolescent patients, were used to assess the symptoms of eating disorders, depression, obsessions and compulsions, respectively.

All patients were enrolled in a behaviorally oriented nutritional rehabilitation program. The daily caloric intake was 2,000–2,500 kcal and increased gradually to 3,500–4,000 kcal depending on weight gain (1.0–1.5 kg per week). Group and family therapy were available. The patients with acute symptoms such as severe agitation, anxiety, or insomnia received medication, mostly hydroxyzine, benzodiazepines, or small doses of atypical antipsychotics, only temporarily.

The methodology of this study is similar to other studies previously published by our team (36, 37). Written informed consent was obtained from all participants and their guardians. The research protocol was approved by the Bioethics Committee of Poznan University of Medical Sciences (1029/13). All procedures were conducted in accordance with the 1964 Helsinki Declaration.

### Biochemical Analysis

Venous blood was collected upon morning admission (7–8 am) from overnight fasting malnourished AN patients (AN-T1) and again on the day of discharge, after partial weight normalization (AN-T2). Serum was immediately separated from the blood by centrifugation at 1,000 × g for 15 min at 4°C, aliquoted into Eppendorf tubes, frozen at -70°C, and assayed afterward.

A quantitative assay of OXY was performed using a commercial immunoenzymatic test (General Oxytocin Elisa kit cat no. E9802Ge, EIAab Science Inc, Wuchan, Hubei, China), following the manufacturer’s instructions. The measurement range of the kit was 32.1–2,000 pg/ml. The minimum detectable dose of general OXY is typically less than 0.39 pg/ml. Optical density was read *via* a spectrophotometric plate reader (Biochrom Asys UVM 340 Microplate Reader) at a wavelength of 450 nm ± 10 nm. Every assay was repeated twice, and the mean value of the two assays was used for statistical evaluation. A four-parameter algorithm (four-parameter logistic) was used to assay concentration in the tested samples. The intra-assay coefficient of variation was <4.5%, whereas the inter-assay coefficient of variation was <7.5%.

Enzyme-linked immunosorbent assay analyses were performed using BDNF DuoSet (cat. No DY 248) and TrkB DuoSet (cat. No DY 397-5) ELISA Development Kit (R&D System, Minneapolis, MN, USA) according to the manufacturer’s instructions. All samples and standards were run in duplicates. Standard curves ranged from 1,500–23.4 pg/ml (for BDNF) to 3,000–46.8 pg/ml (for TrkB). Intra-assay and inter-assay variability were <5% and <10% coefficient of variation (CV), respectively.

### Statistical Analysis

Results were analyzed using the SPSS 21 statistical package. The data were reported as mean ± standard deviation (SD). Results are also presented as boxplots where the dark line in the middle of the box is the median, the bottom of the box indicates the 25th percentile, and the top the 75th percentile with whiskers extended to the minimum and maximum values. The distribution of results was tested for normal distribution using the Shapiro–Wilk tests. For variables with normal distribution (age CG, weight CG, BMI AN-T1, BMI CG, %IBW CG, %IBW AN-T1, OXY AN-T1, OXY AN-T2, TrKB CG), the significance of differences was assessed using the Student’s t-test for dependent (AN-T1 vs. AN-T2) and independent groups (AN-T1 vs. CG and AN-T2 vs. CG). The other variables were analyzed using the Mann–Whitney test for independent groups (AN-T1 vs CG and AN-T2 vs. CG) and the Wilcoxon test for dependent groups (AN-T1 vs AN-T2). Correlations were tested using the Spearman tests. All analyses were two-sided, and p-values ≤0.05 were considered significant. The *post hoc* power was calculated using GPower program (38).

## Results

### Demographic and Clinical Characteristics

The demographic characteristics of all participants are presented in **Table 1**. Studied groups did not significantly differ in age–AN patients were 15.76 ± 2.15 years old and the CG was 15.38± 1.47 years old (p < 0.360) or height–AN group was 1.63 ± 0.06 m and the CG was 1.64 ± 0.05 m (p < 0.281). Body weight, BMI, and %IBW were significantly lower in the AN-T1 than in the AN-T2 and CG.

**Table 1:** Anthropometric and psychometric data, in the adolescent anorexic inpatients in acute stage of the disease (AN-T1) as well as after partial weight recovery (AN-T2), and in the control group (CG).

	AN-T1	AN-T2	CG	AN-T1 vs CG	AN-T1 vs AN-T2	AN-T2 vs CG
					p<	
Age (years)	15.76±2.15		15.38±1.47	0.360		
Height (m)	1.63±0.06		1.64±0.05	0.281		
Weight (kg)	37.76±4.42	45.83±4.64	53.73±11.22	0.000	0.000	0.001
BMI (kg/m2)	14.03±1.58	17.19±1.25	19.81±3.85	0.000	0.000	0.001
%IBW	52.63±8.35	65.79±6.15	74.99±14.24	0.000	0.001	0.003
HDRS	12.80±7.09	5.46±4.94	3.07±5.08	0.000	0.000	0.080
BDI	16.24±12.25	9.81±10.39	3.53±4.24	0.000	0.000	0.004
EAT-26	25.87±20.15	15.35±19.20	5.20±5.55	0.000	0.067	0.007
YBOCS	10.10±7.84	6.84 ± 8.66	3.53±4.24	0.000	0.158	0.071

The AN-T1 group obtained statistically significant higher results on the HDRS than the AN-T2 group (p < 0.000) and CG (p < 0.000), but there was no statistically significant difference between the AN-T2 group and CG (p < 0.080). The AN-T2 group scored statistically significantly lower in BDI than the AN-T1 group (p < 0.000) but still significantly higher than the CG (p < 0.004). There were no statistically significant differences in EAT-26 between the AN-T1 and AN-T2 group (p < 0.067), but the results in the AN-T1 and AN-T2 group were statistically significantly higher than in the CG (p < 0.000 and p < 0.007, respectively). The results from the YBOCS were higher in the AN-T1 group than in the CG (p < 0.000), but there were no statistically significant differences between the AN-T2 group and CG (p < 0.158), as well as the AN-T1 and AN-T2 group (p < 0.158).

### Serum Levels of OXY, BDNF, and TrkB

**Figure 1:**
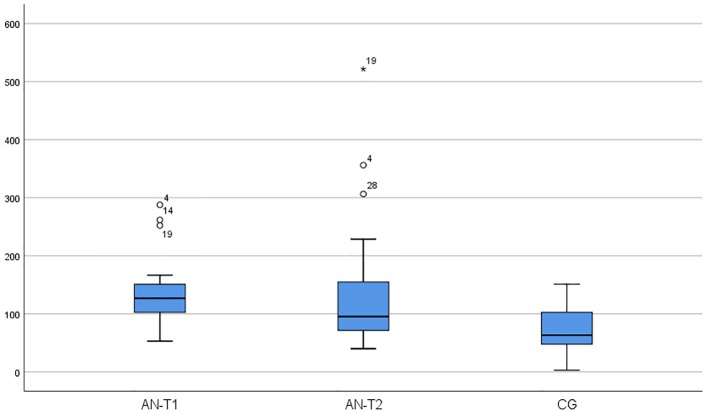
Serum oxytocin (OXY) concentration in the adolescent anorexic inpatients in acute stage of the disease (AN-T1) as well as after partial weight normalization (AN-T2), and in the control group (CG).

**Figure 2:**
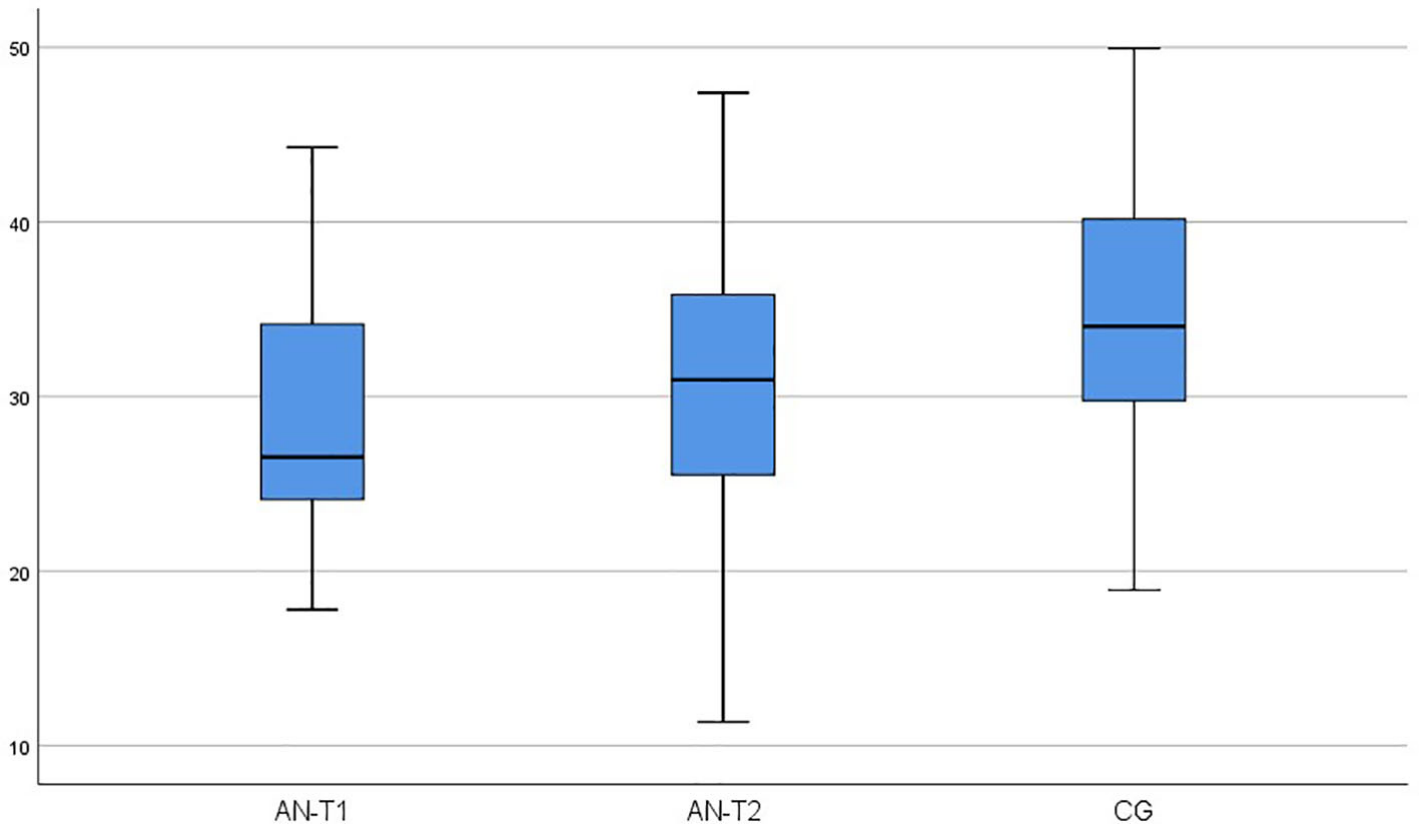
Serum brain-derived neurotrophic factor (BDNF) concentration in adolescent anorexic inpatients in the acute stage of the disease (AN-T1) as well as after partial weight normalization (AN-T2), and in the control group (CG).

**Figure 3:**
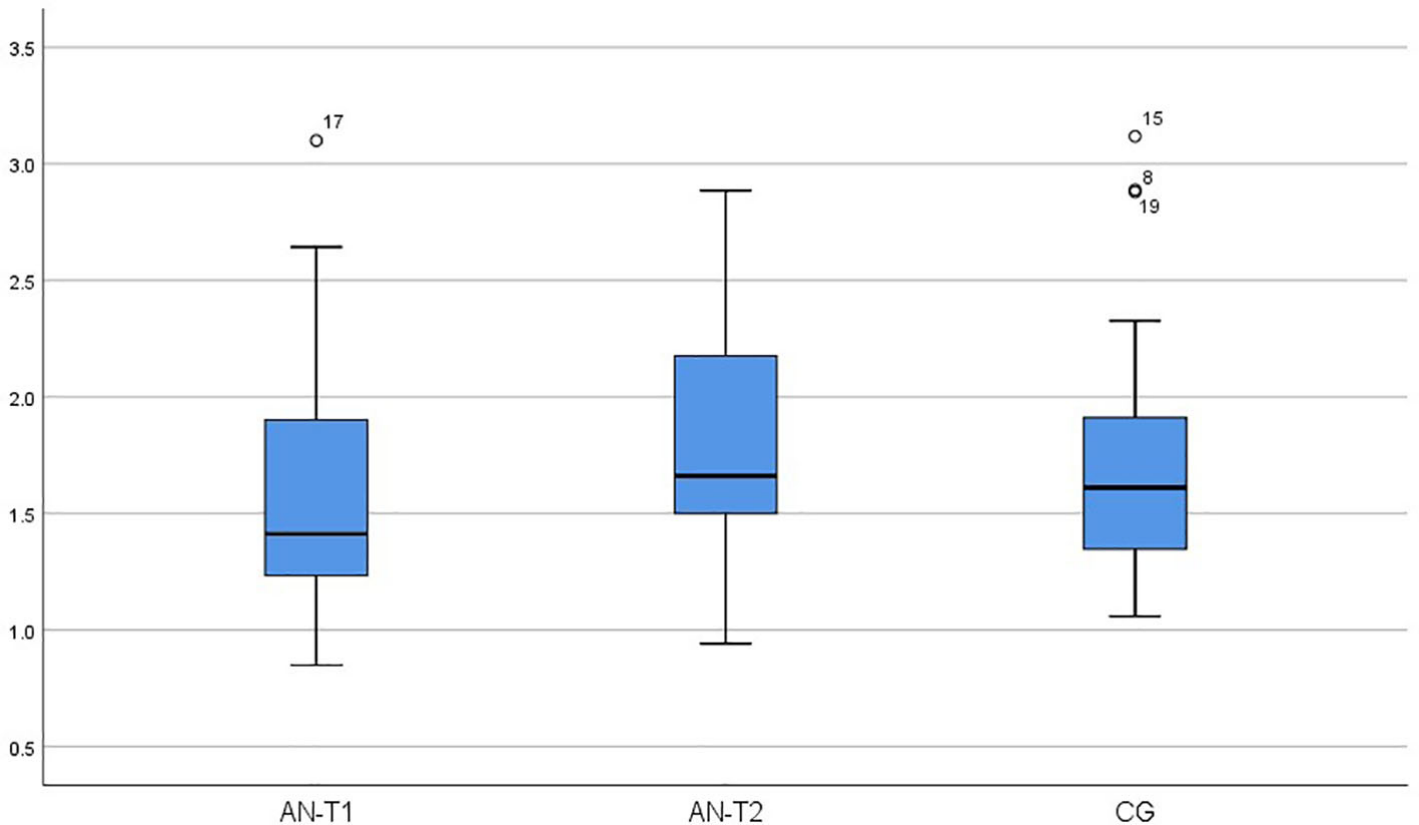
Serum tropomyosin-related kinase B receptor (TrkB) concentration in adolescent anorexic inpatients in the acute stage of the disease (AN-T1) as well as after partial weight normalization (AN-T2), and in the control group (CG).

As presented in **Table 2**, serum OXY concentrations were significantly higher in the AN-T1 and AN-T2 group than the CG (p < 0.000 and p < 0.008 respectively). In AN patients, the OXY levels remained unchanged; there were no statistically significant differences between the AN-T1 and AN-T2 group (p < 0.973) (**Figure 1**). The mean serum BDNF concentrations were statistically significantly higher in the CG than in the AN-T1 group (p < 0.001) and AN-T2 group (p < 0.047). There were no statistically significant differences in the BDNF levels between the AN-T1 and AN-T2 group (p < 0.057), but the tendency to increase with body weight normalization was observed (**Figure 2**). Moreover, there were no statistically significant differences in TrkB concentrations in the AN-T1 group and CG (p < 0.584) or AN-T2 group and the CG (p < 0.147). However there was a statistically significant difference between the AN-T1 and AN-T2 group (p < 0.000)—the TrKB concentrations increased with body weight normalization to the level higher than in the CG (**Figure 3**).

**Table 2:** The serum oxytocin (OXY), brain-derived neurotrophic factor (BDNF), and tropomyosin-related kinase B receptor (TrkB) levels in the adolescent anorexic inpatients in the acute stage of the disease (AN-T1) as well as after partial weight recovery (AN-T2), and in the control group (CG).

	OXY (pg/ml)	BDNF (ng/ml)	TrkB (ng/ml)
AN-T1	127.46 ± 58.68	28.66 ± 6.70	1.67 ± 0.54
AN-T2	127.90 ± 106.13	30.75 ± 8.34	1.93 ± 0.53
CG	70.72 ± 39.75	34.66 ± 7.40	1.74 ± 0.53
AN-T1 vs. CG			
df	58	67	67
p	0.000	0.001	0.584
t	4.385	-3.521	-5.551
md (95% cl)	56.740 (30.760–82.719)	-5.998 (-9.398–(-2.597)	-0.071 (-0.330–0.187)
power	0.99	0.98	0.09
AN-T1 vs. AN-T2			
df	29	38	38
p	0.973	0.057	0.000
t	-0.034	-1.965	-4.102
md (95% cl)	-0.447 (-27.274–26.379)	-2.087 (-4.239–0.063)	-0.260 (-0.388–(-0.131)
power	0.05	0.30	0.72
AN-T2 vs. CG			
df	58	67	67
p	0.008	0.047	0.147
t	2.764	-2.025	1.468
md (95% cl)	57.187 (15.769–98.604)	-3.910 (-7.764–(-0.056)	0.188 (-0.067–0.445)
power	0.83	0.55	0.31

### Correlations Between Analyzed Variables

A statistically significant negative correlation between BDNF and BMI was found for the AN-T1 group (p < 0.036; r = −0.372) but not the AN-T2 group. A statistically significant negative correlation between BDNF and EAT-26 (p < 0.042; r = −0.292) was obtained. TrkB showed no statistically significant correlation with any of the analyzed variables in the AN-T1 or AN-T2 groups. However, a positive correlation between TrkB and BMI was found in AN (p < 0.000; r = 0,369). There were no statistically significant correlations between OXY and BMI, HDRS, BDI, EAT-26, or YBOCS in any of the patient groups or CG.

## Discussion

The adolescent inpatients with the restrictive type of AN included in the presented study were hospitalized for about 11 weeks and gained, on average, 8 kg. After partial weight recovery, they showed improvement in obsessive-compulsive and depressive symptoms assessed by a psychiatrist—the AN-T2 group and the CG showed no statistically significant differences in HDRS and YBOCS. Conversely, the AN-T2 group still presented higher than the healthy controls, score in self-reported depression (BDI) and eating disorder (EAT-26) scales. These results are partially in line with previous studies (39), which showed that weight restoration alone is insufficient for long-term recovery (40, 41). Many psychopathological symptoms and biological abnormalities, including alteration in peripheral and central neuropeptides, still occur long after weight normalization and may affect disease course and prognosis (42).

The existing literature examining OXY in AN subjects is small (43). To the best of our knowledge, none of the studies assessed levels of OXY in AN patients below 18 years old, though the highest incidence rate subgroup for AN includes girls aged 15–19 years old and accounts for about 40% of all identified cases. Results from studies conducted in adult AN patients are conflicting. In one study, no differences in basal OXY levels between AN and control groups were demonstrated (44). However, in most of the investigations, it was found that OXY levels were decreased in AN adult patients in serum (17, 45), as well as in CSF (not in binge-purge subtypes) (46). Moreover, postprandial OXY level was also discovered to be elevated in AN patients (47). The results of the present research are contradictory—serum OXY levels were higher in the AN-T1 and AN-T2 than in the healthy controls. Moreover, the OXY level did not normalize with weight gain, and after partial weight normalization remained higher than in the CG. A high level of anorexigenic OXY might suggest disturbances in transferring information about the metabolic status and be co-responsible for maintaining low body weight. It could also be an adaptive mechanism to reduce the level of cortisol and anxiety. Previous studies have shown that OXY reduces the level of adrenocorticotropic hormone and cortisol under basal and stress conditions (48) and acts as an antianxiety factor.

The link between OXY levels and psychopathology in AN has also been explored. Studies in AN women have identified an association between genetic variation in the OXY receptor and eating disorders, thoughts and behaviors, and food preoccupation (49, 50). Moreover, basal OXY levels in patients after partial weight recovery, but not in the acute stage, were negatively associated with disordered eating psychopathology and anxiety symptoms (45), but postprandial OXY levels were positively associated with disordered eating, anxiety, and depressive symptoms (47, 51). In the present study, we did not find any correlation of OXY with symptoms of eating disorders, depression, obsessions or compulsions.

Using various methodological approaches might be the simplest explanation for the inconsistency in the results of studies. However, it has been postulated that OXY levels might be not only sex-dependent but also age-dependent (52, 53) and this could justify conflicting results as well. Different actions of OXY related to age has been suggested in both animal (54) and human studies (55). Intranasal administration of OXY can lead to a reduction in body weight when given to obese adults but not to obese children; thus, it was postulated that young individuals might respond differently to OXY than older ones (56). Therefore, it is necessary to study OXY levels in adolescent girls because, in many ways, it appears to be a critical period for biological and psychological development.

BDNF levels have been intensively studied in eating disorders, mostly in small groups of adult patients. In the previous investigations, serum BDNF levels were decreased in the acute stage of AN (57–61), what was also confirmed in a meta-analysis (62). The results were opposite in only one study, probably due to heterogeneity of the included patients and the fact that plasma and not serum was assayed (63). After partial or full weight normalization, BDNF levels were increased (27, 59, 64, 65), unchanged (58), or even decreased (65, 66). In the present study in the acute stage of the disease, BDNF levels were reduced compared to the healthy controls and tend to normalize with weight gain. Moreover, a negative correlation between BDNF and BMI was found in the AN-T1 patients but not in AN-T2. There was also a positive correlation between BMI and TrkB in AN patients. Previously, either no association of BDNF with BMI (58, 59) or a positive relationship (60, 61, 66) was shown in the analysis of all patients regardless of body weight and the stage of the disease. Lower levels of BDNF could be an adaptive mechanism to promote food intake in chronic starvation. However, its role in non-homeostatic regulation of food intake could also be important in terms of an explanation. This was shown in genetic studies where the Val66Met BDNF polymorphism was associated not only with BMI (67) but also with a higher reward value of starvation in AN (68).

Moreover, negative correlations between the severity of symptoms of eating disorders measured with EAT-26 in AN patients were found (p < 0.042; r = -0.292). This is contradictory with other results where Eating Disorder Examination Questionnaire (EDEQ) or Eating Disorders Inventory two (EDI-2) were used and no correlations with eating disorder symptoms were found (59, 61). We did not see an association between BDNF and depressive symptoms measured with HAM or BDI or obsession and compulsions measured with YBOCS. These findings confirmed previous studies where BDNF did not correlate with the severity of the depressive (57, 60, 61) or obsessive-compulsive symptoms (59) in AN patients. We speculate that BDNF could be related to aberrant eating behaviors occurring in AN.

The authors of the presented study are aware of its limitations and recall the most important of them. Only weight-recovered patients were included in the study, and it would be relevant to include also those who did not reach appropriate body weight or discharge themselves against medical advice. The OXY, BDNF, and TrkB levels should be examined several times at the different time points as well as after long-term weight restoration what could help draw the final conclusions.

### Conclusions

BDNF serum concentration was lower in the malnourished AN patients and tended to normalize its level with weight gain. The serum of OXY was higher in adolescent inpatients with AN and did not normalize its level; thus, it could contribute to persistent psychopathology after partial weight recovery from AN.

BDNF levels correlate with the severity of eating disorder symptomatology and, thus, could be related to aberrant eating behaviors occurring in AN. Our results do not support a role for serum level BDNF in the modulation of depressive and obsessive-compulsive symptoms.

## Data Availability Statement

The datasets generated for this study are available on request to the corresponding author.

## Ethics Statement

The studies involving human participants were reviewed and approved by Bioethics Committee of Poznan University of Medical Sciences (1029/13). Written informed consent to participate in this study was provided by the participants’ legal guardian/next of kin.

## Author Contributions

MT-N, AS, and FR contributed conception and design of the study. MD-W, AD and EP organized the database. MT-N and MS performed the statistical analysis. MD-W and MS performed biochemical analysis. All authors contributed to manuscript revision, read and approved the submitted version.

## Conflict of Interest

The authors declare that the research was conducted in the absence of any commercial or financial relationships that could be construed as a potential conflict of interest.
